# Arterial stiffness association with chronic inflammatory disorders in the UK Biobank study

**DOI:** 10.1136/heartjnl-2017-312610

**Published:** 2018-01-04

**Authors:** Alex Dregan

**Affiliations:** 1 School of Population Health Sciences, King’s College London, London, UK; 2 NIHR, Biomedical Research Centre at Guy’s and St Thomas NHS Foundation Trust, London, UK

**Keywords:** inflammatory markers, systemic inflammatory diseases, epidemiology, aortic and arterial disease, cardiac imaging and diagnostics

## Abstract

**Objective:**

The present study tested the hypothesis that arterial stiffness will be elevated across overall and specific inflammatory disorders compared with an inflammation-free comparison group.

**Methods:**

Adults (n=171 125) aged 40–70 years from the UK Biobank who were cardiovascular disease (CVD) free and who had their arterial stiffness assessed at the time of study recruitment between 2006 and 2010 were included. The main exposure was represented by a global measure of chronic inflammatory disorders. Two inflammatory biomarker measures (eg, leucocytes count, granulocytes count) were included as markers of inflammation severity. The arterial stiffness index assessed by a non-invasive technique represented the study primary outcome measure.

**Results:**

A total of 5976 (3%) participants diagnosed with inflammatory disorders and 165 149 participants without an inflammatory disorder had data on arterial stiffness. Adjusted linear regression analyses revealed a 14% increment in mean arterial stiffness for chronic inflammatory disorders (beta coefficient (β) 1.14, 95% CI 1.05 to 1.24, P=0.002) compared with no chronic inflammatory disorder. Arterial stiffness tended to increase (P value=0.031) with tertiles of leucocytes and granulocytes count. For instance, mean arterial stiffness values increased from 1.11 (95% CI 0.96 to 1.29) in the first tertile to 1.17 (95% CI 1.02 to 1.34) in the second tertile, and 1.21 (95% CI 1.05 to 1.39) in the third tertile of leucocytes count. There was evidence for similar associations with some of the most common individual inflammatory disorders, including psoriasis and rheumatoid arthritis.

**Conclusion:**

Arterial stiffness was associated with multiple chronic inflammatory disorders. An increasing trend in mean arterial stiffness was also documented with increasing tertiles of different inflammatory biomarkers. Future studies are needed to investigate the discriminant value of arterial stiffness to predict major CVD events within various inflammatory disorders.

Cardiovascular diseases (CVD) represent a major burden among people diagnosed with chronic inflammatory disorders. For instance, several inflammatory disorders (eg, rheumatoid arthritis (RA), psoriasis and inflammatory bowel disorders (IBD)) were found to confer a 50% increased risk of major vascular events (eg, myocardial infarction (MI), stroke) compared with the general population.^1–3^ In addition to traditional vascular risk factors (eg, hypertension, dyslipidaemia, obesity, smoking), inflammation has been documented as a major determinant of CVD risk across diverse inflammatory disorders.^1^ These findings emphasise the importance of identifying the pathways through which chronic inflammation may lead to early CVD pathogenesis. Studies with small (mainly hospital-based) samples point towards a potential association between inflammation with arterial stiffness, an independent predictor of future CVD events.^4–6^ Research in this area remains primarily exploratory, however, with studies employing diverse non-invasive measures of arterial stiffness (eg, pulse wave velocity (PWV), augmentation index, stiffness index (SI)) across different populations, making it difficult to draw robust overall conclusions.^7–9^ The UK Biobank study includes several physiological and imaging techniques that may facilitate early identification of future risk of CVDs. For instance, the study includes an SI measure as a surrogate marker for subclinical atherosclerosis. While studies with small healthy populations indicate moderate correlation between the SI and the PWV,^10^ no studies have evaluated the prognostic value of SI within diverse inflammatory disorders. Recently, the study added data on leucocytes count that have been used as markers of inflammation severity. It has been documented, for instance, that leucocytes release cytokines, triggering further macrophage recruitment and the proliferation of smooth muscle cells within the vascular wall.^11^ In addition, protease secretion leads to endothelial damage of the coronary vessels, exposing thrombogenic collagen and predisposing the vessels to thrombus formation. Phagocytes release myeloperoxidase, which generates reactive oxygen species that are involved in the generation and progression of atherosclerosis and that contribute to the development of plaque instability in acute MI.^11^ Whether chronic inflammation is associated with a surrogate marker of arterial stiffness is not clearly established. The present study aimed to address this concern within a large community-based sample of adults aged 40–69 years. The study’s main hypothesis was that arterial stiffness would be more common among inflammatory disorders relative to people in the general population, and that this association would vary with disease severity.

## Methods

### Data

The data for the present study come from the UK Biobank, a large population-based prospective study developed to facilitate detailed investigations about the determinants of diseases at population level. The UK Biobank collects detailed data from over 500 000 participants aged 40–69, including lifestyle, demographics, clinical diagnoses, treatment, lab tests (ie, biomarkers), imaging and genotype information.^12^ A more detailed description of the UK Biobank data is provided elsewhere.^12^

The present study was restricted to a subset of study participants (n=171 125) with measured arterial stiffness at baseline. Participants diagnosed with RA, systemic lupus erythematosus (SLE), Sjogren syndrome, psoriasis, ankylosing spondylitis (AS), systemic vasculitis or IBD represented the exposed group. Participants free of these diagnoses represent the comparison group. Following Shen *et al* 13 the study excluded participants with a history of CVDs (including coronary heart disease, stroke and peripheral arterial disease) or type II diabetes. Because arterial stiffness represents a blood pressure independent predictor of CVD events,^14^ patients with previous hypertension have been included in the analyses. This procedure allowed to account for the potential modulatory role of antihypertensive therapies on arterial stiffness.^15^ All participants provided written informed consent.

### Arterial stiffness outcomes

A photoplethysmograph transducer was placed on the index finger of the participant’s dominant hand and used to calculate an arterial SI. The SI was assessed as the height of the participants divided by the time between the first (systolic) and second (diastolic) wave peaks, and was expressed in metres per second (m/s). SI is a clinical marker of larger artery stiffness and has been found to be moderately correlated with PWV—the gold standard measure of arterial stiffness.^10^ A higher SI is considered indicative of stiffer arteries.

### Exposures

The study’s main exposure was a global measure of chronic inflammatory disorders, including RA, psoriasis, IBD, SLE, Sjogren syndrome, systemic vasculitis and AS. These measures were developed from participants’ self-reports and were recorded according to the *International Classification of Diseases, 10th Revision* criteria. To explore potential variation within specific inflammatory disorders, separate binary variables (yes/no) were developed for the most common inflammatory disorders, specifically RA, IBD and psoriasis. Leucocytes count was used to categorise participants into tertiles of *inflammation disease severity*. A composite granulocytes count-based inflammation severity measure was also developed. This measure classified participants into tertiles based on the average count of neutrophil, basophil and eosinophil biomarkers. An inflammation *duration* variable, which classified participants into tertiles of disorder duration, was used for sensitivity analyses.

### Covariates

The study covariates included sociodemographic characteristics, specifically age (continuous measure), gender (female vs male), ethnicity (White, Black, Asian, Other) and deprivation. Deprivation was based on Townsend deprivation indices derived from aggregated data on car ownership, household overcrowding, owner occupation and unemployment (higher scores represent higher degree of deprivation). The study also adjusted for traditional vascular risk factors, including smoking (never, ex, current smoker), hypertension (yes/no), hypercholesterolaemia (yes/no) and body mass index (BMI; kg/m^2^). Antihypertensive and lipid-lowering drugs (yes/no) were also included as covariates. Non-steroidal anti-inflammatory drugs (NSAID) and corticosteroid drugs were included as separate binary covariates (they may have different impact on leucocytes count) due to their association with increased CVD risk.^16^ Sensitivity analyses also adjusted for disease-modifying antirheumatic drug (DMARD) prescribing.

### Statistical analysis

Descriptive analyses (eg, frequencies, means) were used to describe baseline characteristics among participants diagnosed with inflammatory disorders and the comparison group. The associations between inflammatory disorders with the SI were estimated using multivariable linear regression analyses with robust SE. Separate estimation models were performed for overall and specific inflammatory disorder (eg, RA, psoriasis and IBD). All models adjusted for age, sex, deprivation, BMI, smoking, ethnicity, previous diagnoses of hypertension and hypercholesterolaemia, anti-inflammatory drugs, as well as antihypertensive and lipid-lowering medications. Analyses considering multiple imputation provided similar estimates to the main estimation models and only the latter are presented here. To test for potential dose–response associations, linear regression was used to compare inflammatory participants in different leucocytes count tertiles to the reference group of no chronic inflammatory disorder.^1^ Sensitivity analyses stratified by inflammation disorder were also performed. Additional sensitivity analyses exploring the association between inflammatory disorder duration with the SI measure and adjusting for DMARDs were performed. The study did not adjust for multiple comparisons as it could increase the risk of type II errors.^17^ Results are reported as β estimates with 95% CIs. All analyses were conducted using STATA V.14 software (*regress* command with the *robus*t option), using a P value of 0.05 as the threshold for statistical significance.

## Results

After excluding participants with a diagnosis of CVD or diabetes prior to SI assessment, the analyses included 5976 participants diagnosed with an inflammatory disorder and 165 149 participants without an inflammatory disorder. The most common disorder was psoriasis (n=2019), followed by RA (n=1679) and IBD (n=1392). For all groups, the distribution of age was similar (table 1). Women were over-represented across all conditions, except psoriasis (48%). Participants diagnosed with chronic inflammatory disorders presented with higher prescribing rates of NSAIDs (25%), corticosteroids (14%), DMARDs (18%) and antihypertensive (21%) drugs relative to the comparison group (18%, 3%, 0% and 16%, respectively).

**Table 1:** Participants’ characteristics at baseline assessment

	All (5976)	RA (n=1672)	Psoriasis (2091)	IBD (1392)	Unexposed (165 149)
Age, mean (SD)	58 (8)	59 (7)	57 (8)	57 (8)	57 (8)
Gender, female	3486 (58)	1173 (70)	1013 (48)	783 (56)	93 312 (55)
Deprivation, mean (SD)	−1.17 (3)	−1.06 (3)	−1.10 (3)	−1.31 (3)	−1.21 (3)
Ethnicity					
White	5726 (94)	1534 (92)	1988 (95)	1320 (95)	150 412 (92)
Asian	183 (3)	68 (4)	56 (3)	37 (3)	5368 (3)
Black	76 (1)	31 (2)	8 (1)	11 (1)	4913 (3)
Other	100 (2)	34 (2)	31 (1)	19 (1)	4024 (2)
Smoker					
Never	2921 (49)	827 (50)	979 (47)	683 (49)	93 508 (57)
Ex	2344 (39)	654 (39)	805 (39)	586 (42)	54 919 (33)
Current	686 (12)	183 (11)	298 (14)	120 (9)	15 813 (10)
BMI, mean (SD), kg/m^2^	27.65 (5)	27.72 (5)	28.11 (5)	26.98 (4)	27.24 (5)
Antihypertensive drugs	1292 (21)	390 (23)	414 (20)	257 (18)	27 127 (16)
Statins	748 (12)	238 (14)	254 (12)	142 (10)	17 619 (11)
NSAIDs	1535 (25)	600 (36)	451 (22)	211 (15)	29 636 (18)
Corticosteroids	866 (14)	266 (13)	166 (12)	108 (8)	4297 (3)
DMARDs	1125 (18)	694 (42)	168 (8)	162 (12)	301 (0)
Hypertension	1830 (30)	542 (32)	611 (29)	349 (25)	41 984 (25)
Hypercholesterolemia	809 (13)	229 (14)	305 (15)	136 (10)	20 726 (13)

Figures are numbers and percentages unless otherwise specified.

DMARD, disease-modifying antirheumatic drug; IBD, inflammatory bowel disorder; NSAID, non-steroidal anti-inflammatory drug; RA, rheumatoid arthritis.

**Figure 1:**
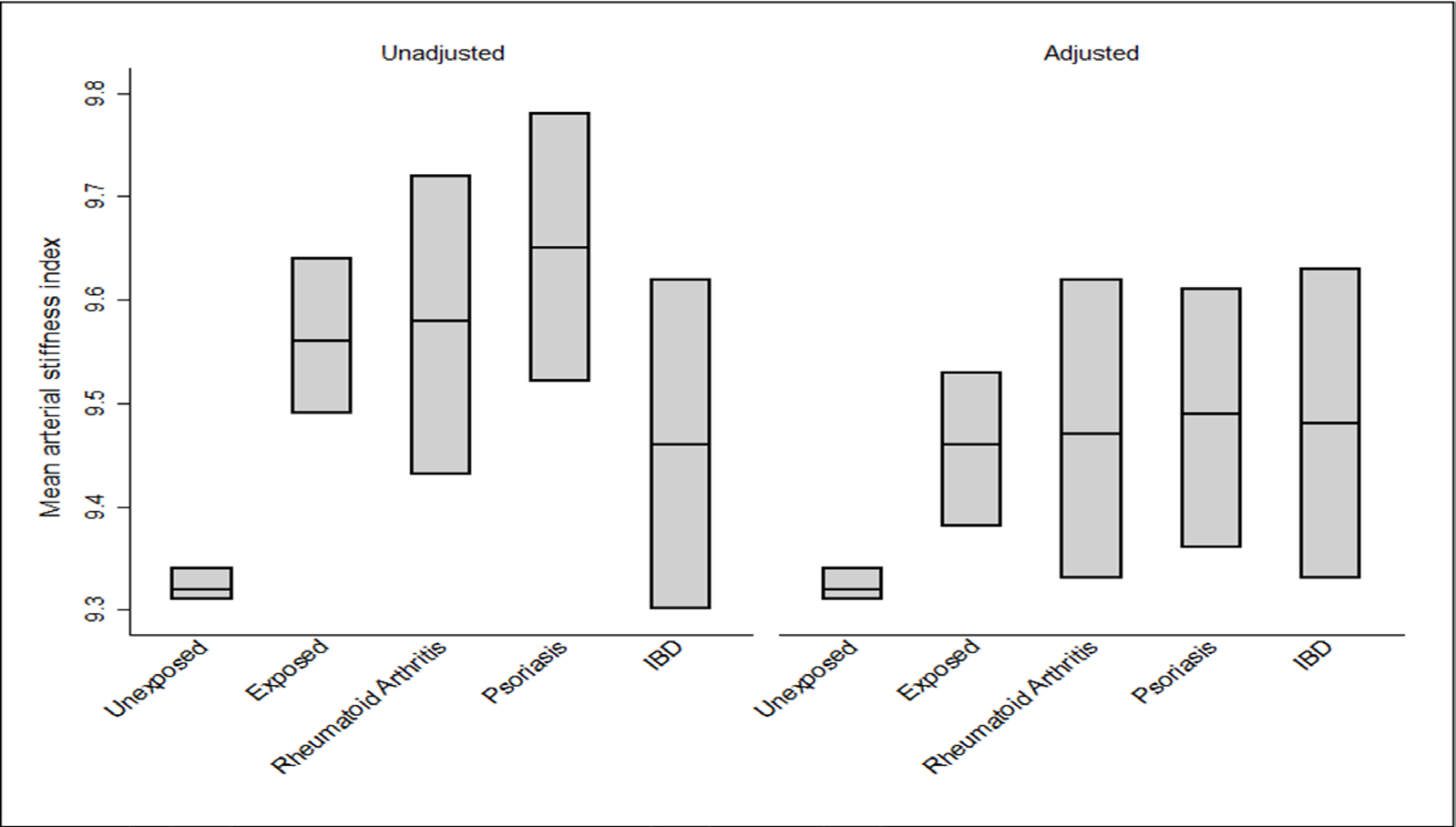
Unadjusted and adjusted mean values for the arterial stiffness index across the study groups. IBD, inflammatory bowel disorder.

Figure 1 (see online supplementary figure S1 for data on all inflammatory disorders) illustrates the adjusted and unadjusted mean SI values for chronic inflammatory participants and their comparison group. Mean SI values were higher in the group (9.46, 95% CI 9.38 to 9.53) relative to the comparison group (9.32, 95% CI 9.31 to 9.34). Also, RA (9.47, 95% CI 9.33 to 9.62), IBD (9.48, 95% CI 9.33 to 9.63) and psoriasis (9.49, 95% CI 9.36 to 9.61) disorders presented with the highest mean values for the SI.

10.1136/heartjnl-2017-312610.supp1 Supplementary file 1

Multivariable linear regression analyses (table 2) revealed that chronic inflammatory disorders were associated with 14% increase in mean arterial stiffness value (β=1.14, 95% CI 1.05 to 1.25, P=0.002) compared with no chronic inflammatory disorder. Mean SI values increased gradually with tertiles of the composite biomarkers of inflammation. For instance, the SI increased from 11% in the first tertile (95% CI 0.96 to 1.29, P=0.162) to 17% in the second tertile (95% CI 1.02 to 1.34, P=0.029), and 21% in the third tertile (1.21, 95% CI 1.05 to 1.39, P=0.009) of leucocytes count. Postestimation analyses revealed that the differences in mean arterial stiffness between the three leucocyte tertiles were statistically significant (P value=0.031). A similar pattern emerged with regard to the composite granulocytes count measure; however, the association was statistically significant only at the highest tertile of granulocytes count (1.23, 95% CI 1.06 to 1.42, P=0.005).

**Table 2:** Exponentiated coefficients and associated 95% CI for the association between overall chronic inflammatory disorders and inflammation severity with the arterial stiffness index

	Unadjusted model	P value	Fully adjusted model	P value
	β (95% CI)		β (95% CI)	
Overall inflammatory disorders	1.29 (1.19 to 1.40)	0.001	1.14 (1.05 to 1.24)	0.002
**Severity**				
Leucocytes count, range ×10^9^ cells/L (minimum-maximum)				
First tertile (0.01–6.11)	1.08 (0.93 to 1.25)	0.294	1.11 (0.96 to 1.29)	0.162
Second tertile (6.12–7.69)	1.32 (1.14 to 1.51)	0.001	1.17 (1.02 to 1.34)	0.029
Third tertile (7.7–45.91)	1.57 (1.36 to 1.81)	0.001	1.21 (1.05 to 1.39)	0.009
Granulocytes count, range ×10^9^ cells/L (minimum-maximum)				
First tertile (0.01–1.31)	1.06 (0.92 to 1.23)	0.414	1.12 (0.97 to 1.30)	0.114
Second tertile (1.31–1.73)	1.26 (1.10 to 1.44)	0.001	1.13 (0.98 to 1.29)	0.084
Third tertile (1.74–6.65)	1.61 (1.39 to 1.86)	0.000	1.23 (1.06 to 1.42)	0.005

β, exponentiated coefficient.

Mean SI was also increased within RA (1.18, 95% CI 1.01 to 1.39, P=0.044) and psoriasis (1.21, 95% CI 1.03 to 1.32, P=0.016), but not IBD (1.14, 95% CI 0.98 to 1.35, P=0.091) disorders (table 3). A statistically significant association between severity of inflammation with mean SI was revealed within both RA and psoriasis disorders. In adjusted analyses, however, the association was statistically significant only at the highest tertile of granulocytes count for psoriasis (1.29, 95% CI 1.03 to 1.60, P<0.001).

**Table 3:** Exponentiated coefficients and associated 95% CIs for the association between specific chronic inflammatory disorders and inflammation severity with the arterial stiffness index

	Rheumatoid arthritis		Psoriasis		Inflammatory bowel disorders	
	Unadjusted β (95% CI)	Adjusted β (95% CI)	Unadjusted β (95% CI)	Adjusted β (95% CI)	Unadjusted β (95% CI)	Adjusted β (95% CI)
Disorder	1.29 (1.09 to 1.52)	1.18 (1.01 to 1.39)	1.39 (1.22 to 1.58)	1.17 (1.03 to 1.32)	1.15 (0.98 to 1.36)	1.15 (0.98 to 1.35)
**Severity**						
Leucocytes count tertiles						
First	1.19 (0.88 to 1.60)	1.20 (0.90 to 1.61)	1.03 (0.82 to 1.29)	1.02 (0.82 to 1.28)	1.08 (0.81 to 1.45)	1.17 (0.88 to 1.55)
Second	1.24 (0.93 to 1.64)	1.16 (0.88 to 1.52)	1.66 (1.33 to 2.08)	1.36 (1.09 to 1.69)	1.24 (0.93 to 1.66)	1.24 (0.94 to 1.65)
Third	1.59 (1.19 to 2.13)	1.25 (0.93 to 1.67)	1.65 (1.32 to 2.07)	1.21 (0.97 to 1.50)	1.18 (0.89 to 1.56)	1.10 (0.83 to 1.45)
Granulocytes count tertiles						
First	1.14 (0.85 to 1.52)	1.23 (0.91 to 1.67)	1.12 (0.89 to 1.42)	1.13 (0.90 to 1.42)	1.14 (0.85 to 1.53)	1.26 (0.94 to 1.69)
Second	1.33 (1.00 to 1.75)	1.24 (0.93 to 1.65)	1.45 (1.16 to 1.81)	1.18 (0.95 to 1.47)	1.19 (0.90 to 1.58)	1.21 (0.92 to 1.59)
Third	1.56 (1.16 to 2.11)	1.31 (0.96 to 1.80)	1.71 (1.37 to 2.15)	1.29 (1.03 to 1.60)	1.19 (0.89 to 1.59)	1.09 (0.82 to 1.45)

β, exponentiated coefficient.

### Sensitivity analyses

Analyses that stratified participants by inflammation disorder revealed a gradual increment in mean SI with higher tertiles of leucocytes count. The increase was statistically significant in the reference group but not in the chronic inflammation group, possibly due to insufficient power for the latter. SI values increased linearly from the first to the third tertile of inflammatory disorder duration (years since diagnosis), being statistically significant at the middle (1.16, 95% CI 1.01 to 1.34, P=0.038) and highest tertiles (1.18, 95% CI 1.03 to 1.35, P=0.019) of disorder duration (online supplementary table S1). A similar pattern was also apparent between the SI with RA and IBD disorder duration. Finally, adjusting for DMARD prescribing did not alter the direction or statistical significance of associations.

10.1136/heartjnl-2017-312610.supp2 Supplementary file 2

## Discussion

Using a large community sample, an overall measure of major chronic inflammatory disorders was associated with higher rates of arterial stiffness. The association appeared to increase with the severity of chronic inflammation, as assessed by a composite measure of inflammatory biomarkers (eg, leucocytes count and granulocytes count). Within each composite measure, for instance, the association with arterial stiffness increased gradually from the first to the third tertile. These findings support the possibility of a dose–response association between inflammation severity with a non-invasive measure of arterial stiffness.

Inflammatory disorder-specific analyses supported a modest association between arterial stiffness with psoriasis and RA disorders, but less so with regard to IBDs. This suggestion was supported by the evidence that the SI increased with each tertile of granulocytes count within the psoriasis disorder. No clear dose–response relationship was observed with regard to the leucocytes count measure, implying that leucocytes count may be an insensitive and non-specific marker of inflammation severity or that the study was insufficiently powered for disorder-specific analyses. The UK Biobank’s intention to incorporate biomarker data on C-reactive protein (CRP) and rheumatoid factor would offer the opportunity to compare the prognostic value of leucocytes count with these established markers of chronic inflammation.

While specific chronic inflammatory disorders may vary with regard to clinical presentation and underlying risk factors,^18^ they all have in common elevated levels of low-grade inflammation. These heterogeneous conditions also present similar rates of cardiovascular complications, including major CVD events and atherosclerosis. These associations may vary with inflammation severity, and the present study findings documented the potential value of using composite measures of markers of chronic inflammation for these purposes. The extent to which these findings are translated to specific chronic inflammatory disorders remains to be determined, however.

A growing number of studies suggest that inflammation could be responsible for part of the excess cardiovascular risk observed in patients with chronic inflammatory diseases. Recently, it was reported that, compared with control subjects, carotid-femoral PWV and augmentation index were significantly higher in patients with chronic inflammation, including inflammatory bowel diseases (IBD), RA, SLE and systemic sclerosis.^19–21^ Moreover, a significant relationship between aortic stiffness and left ventricular systolic and diastolic dysfunction was reported in patients with IBD.^22^ This finding may explain the link between increased aortic stiffness with CVD events in patients with IBD and why these patients present with an increased CVD risk despite a low prevalence of classical cardiovascular risk factors.

Empirical evidence about differences in arterial stiffness between diverse chronic inflammatory disorders and the general population is unavailable, precluding any direct comparison between this study findings with previous research. Nevertheless, the findings for increased arterial stiffness associated with psoriasis and RA disorders are in line with earlier evidence with smaller samples and different surrogate markers of arterial stiffness.^15 23 24^ Booth *et al* 25 found increased arterial stiffness among 31 patients diagnosed with systemic vasculitis, but the association was limited to patients (n=15) with active disease. The current study findings for a statistically significant association between arterial stiffness with the highest tertiles of leucocytes and granulocytes count point towards similar evidence across multiple chronic inflammatory disorders. The present study findings appear to support earlier evidence for a lack of association between arterial stiffness with IBDs,^26^ but this suggestion needs to be interpreted within the constraints of insufficient statistical power. Recent meta-analyses^20 21^ reported increased augmentation index and carotid-femoral PWV among patients diagnosed with IBD. The higher, but not statistically significant, SI for IBD condition in this study may be explained by the use of a surrogate marker of arterial stiffness or the remission of disease in participants diagnosed with IBD. These explanations may also account for the lack of association between white blood cells with the SI in this study, which is not in line with suggestions from a recent meta-analysis.^22^

### Strengths and limitations

The present study has several strengths, including large population sample, comprehensive phenotype data, physiological measures of inflammatory biomarkers and objectively assessed arterial stiffness. As with most observational studies, several limitations need consideration in interpreting the study findings. The UK Biobank data are based on self-reported chronic inflammatory disorders, which may increase the risk of misclassification bias. If inflammatory disorder diagnosis was over-reported or under-reported, this bias may slightly attenuate or strengthen the association with arterial stiffness. The prevalence of chronic inflammatory disorders in this study was similar to earlier investigations,^1^ suggesting minimal misclassification bias. The estimation models were limited by the UK Biobank data available at time of study, and direct measures of disease activity (eg, disease activity score) or direct measures of arterial stiffness could not be considered. The present study analyses used composite measures of inflammatory biomarkers (eg, leucocytes, granulocytes), as surrogate markers of inflammation severity. These biomarkers have been documented in the past to differentiate between the preclinical and clinical phases on CVDs.^27^ Plans to include CRP and rheumatoid factor measures into the UK Biobank data would provide for future validation studies with more direct biomarker measurements. As with any observational data, the health survivor bias is a possibility within the UK Biobank. This limitation is of a greater concern for generalising the findings about disease prevalence, and less so about generalising the effect size estimates.^28^ The SI, as assessed by the PulseTrace technique, was found to be only moderately correlated with PWV.^29^ The statistically significant associations and the documented dose–response relationship appear to support a potential role of the SI in the context of inflammatory disorders. This suggestion needs confirmation with future prospective studies, however. Notably, the moderate correlation between SI with PWV observed in Salvi *et al*’s study^29^ could be due to the study insufficient statistical power (n=50) or limited generalisability (just one participant presented a diagnosis of an inflammatory disorder). Information on left ventricular dysfunction, known to be associated with inflammation and CVD risk,^30^ was not available in the data. The linkage of the UK Biobank data with patients’ medical records in the near future would allow for more detailed investigations, including the association between increased arterial stiffness and CVD events in patients diagnosed with chronic inflammation. It would also provide for other investigations, including the impact of anti-tumour necrosis factor therapy on the arterial SI and for validating the diagnosis of chronic inflammatory disorders. The study data were underpowered to provide robust estimates on the association between arterial stiffness with other specific CIs (eg, SLE, AS, systemic vasculitis). Preliminary analyses appeared supportive of increased arterial stiffness within AS and vasculitis disorders, but these findings need validation with larger samples.

## Conclusions

Overall, this study findings support a potential association between markers of chronic inflammation with an estimate of arterial stiffness. The study findings also document a potential role of composite measures of inflammatory biomarkers to discriminate among people diagnosed with inflammatory disorders at different risk of future CVDs. Whether the addition of composite measures of inflammatory biomarkers to traditional CVD risk scores (eg, QRISK2, Framingham) improves the latter discriminant and predictive values within chronic inflammatory disorders awaits further confirmation from prospective studies. Plans to include an MRI-based measure of PWV on the full UK Biobank sample would offer the opportunity to further validate the accuracy of the SI as an estimate of arterial stiffness within the context of chronic inflammatory disorders.

**Key messages**

**What is already known on this subject?**

Patients diagnosed with chronic inflammatory disorders are at increased risk of major cardiovascular events. Strategies to facilitate early identification of subclinical atherosclerosis in chronic inflammation have been proposed over the past years.

**What might this study add?**

Arterial stiffness index was increased among participants diagnosed with chronic inflammatory, including rheumatoid arthritis and psoriasis. The association varied with the distribution of inflammatory biomarkers as markers of inflammation severity.

**How might this impact on clinical practice?**

The stiffness index, a non-invasive measure of arterial stiffness, may provide additional prognostic and discriminatory value for future cardiovascular disease risk prediction within overall and specific chronic inflammatory disorders.
