# Supplementary figures and images for: Arterial stiffness association with chronic inflammatory disorders in the UK Biobank study
Source: Heart. 2018 Jan 4;104(15):1257–62. doi: 10.1136/heartjnl-2017-312610 (PMC6204972; doi:10.1136/heartjnl-2017-312610)

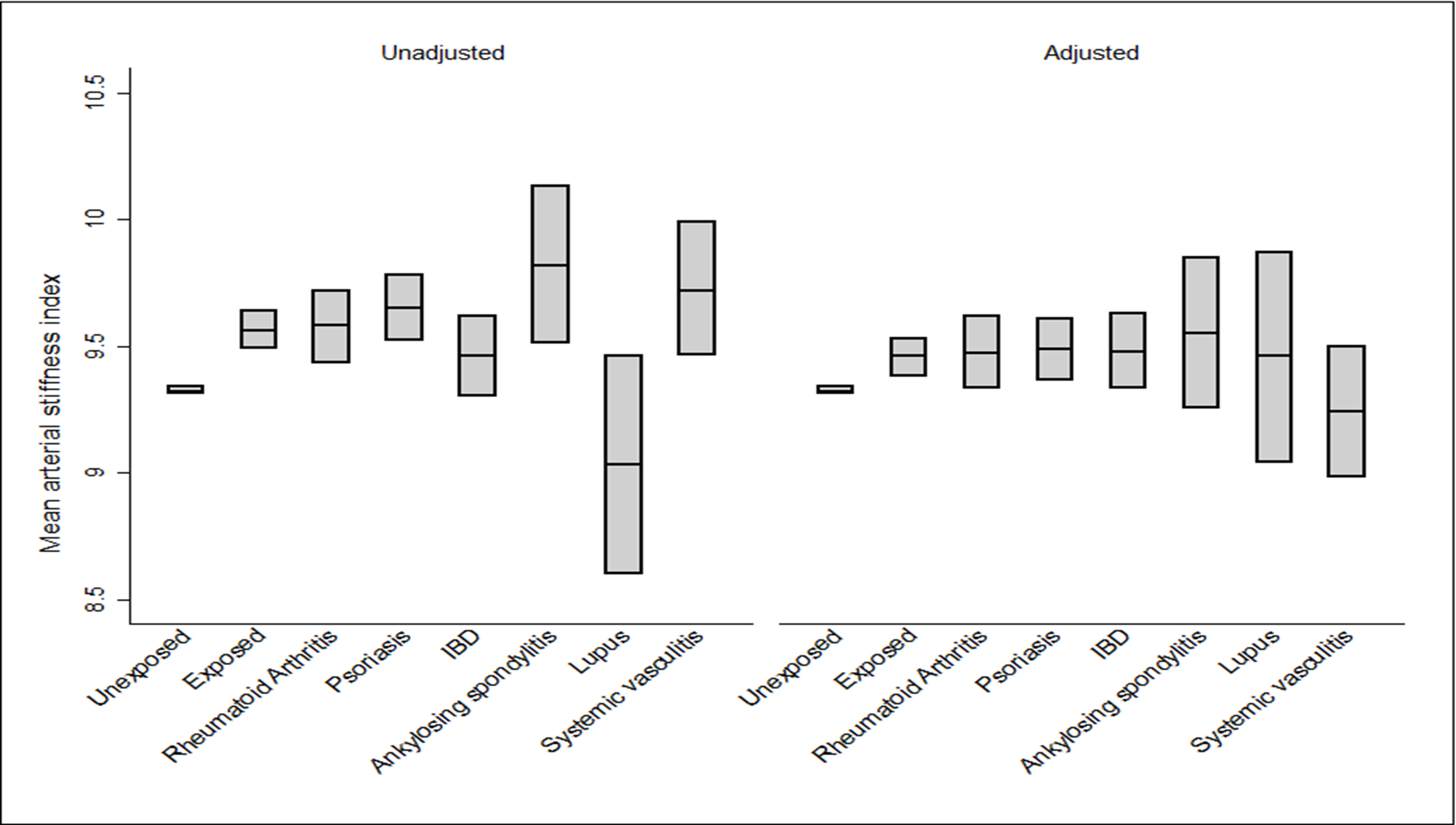

Supplement: Supplementary file 1 [file heartjnl-2017-312610supp001.jpg]
