# Supplementary material for: Arterial stiffness association with chronic inflammatory disorders in the UK Biobank study
Source: Heart. 2018 Jan 4;104(15):1257–62. doi: 10.1136/heartjnl-2017-312610 (PMC6204972; doi:10.1136/heartjnl-2017-312610)
Supplement: Supplementary file 2 [file heartjnl-2017-312610supp002.pdf]

Table S1 Exponentiated coefficients and associated 95% confidence intervals for the association between the duration of chronic inflammatory disorders with the arterial stiffness index

	**Unadjusted model**		**Fully adjusted model**	
	**β (95% CI)**	**p-value**	**β (95% CI)**	**p-value**
Overall inflammatory disorders - Mean years (sd)				
First tertile – 3(3)	1.20(1.05-1.37)	0.009	1.07(0.94-1.22)	0.324
Second tertile -15(4)	1.27(1.10-1.48)	0.001	1.16(1.01-1.34)	0.038
Third tertile – 3(9)	1.34(1.17-1.54)	0.000	1.18(1.03-1.35)	0.019
Rheumatoid Arthritis –				
First tertile – 3(2)	1.10(0.85-1.43)	0.434	1.10(0.85-1.42)	0.458
Second tertile – 11(3)	1.38(1.04-1.85)	0.024	1.13(0.85-1.49)	0.408
Third tertile – 29(10)	1.44(1.06-1.96)	0.018	1.36(1.00-1.85)	0.048
Psoriasis				
First tertile – 6(4)	1.59(1.28-1.96)	0.000	1.31(1.06-1.61)	0.011
Second tertile – 23(6)	1.24(0.98-1.56)	0.063	1.11(0.89-1.38)	0.343
Third tertile – 44(7)	1.29(1.02-1.62)	0.029	1.04(0.83-1.30)	0.697
Inflammatory bowel disorders				
First tertile – 5(3)	1.03(0.79-1.32)	0.821	1.00(0.78-1.28)	0.989
Second tertile – 18(4)	1.02(0.74-1.37)	0.922	1.21(0.90-1.63)	0.215
Third tertile – 35(7)	1.46(1.09-1.94)	0.010	1.26(0.95-1.68)	0.108

Note β – exponentiated coefficients, CI - confidence intervals, sd=standard deviation

Figure S1 Unadjusted and adjusted mean values for the arterial stiffness index across the study groups.
